# Improving Voice Outcomes after Thyroid Surgery and Ultrasound-Guided Ablation Procedures

**DOI:** 10.3389/fsurg.2022.882594

**Published:** 2022-05-04

**Authors:** Pia Pace-Asciak, Jon O. Russell, Ralph P. Tufano

**Affiliations:** ^1^ Department of Otolaryngology – Head and Neck Surgery, University of Toronto, Toronto, Canada; ^2^ Department of Otolaryngology – Head and Neck Surgery, Johns Hopkins University, Baltimore, Maryland, United States; ^3^ Sarasota Memorial Health Care System Multidisciplinary Thyroid and Parathyroid Center, Sarasota, Florida, United States

**Keywords:** thyroid surgery, radiofrequency ablation, dysphonia, voice, vocal cord dysfunction

## Abstract

The field of endocrine surgery has expanded from the traditional open neck approach to include remote access techniques as well as minimally invasive approaches for benign and malignant thyroid nodules. In experienced hands and with careful patient selection, each approach is considered safe, however complications can and do exist. Post-operative dysphonia can have serious consequences to the patient by affecting quality of life and ability to function at work and in daily life. Given the significance of post-procedural dysphonia, we review the surgical and non-surgical techniques for minimizing and treating recurrent laryngeal nerve injury that can be utilized with the traditional open neck approach, remote access thyroidectomy, or minimally invasive thermal ablation.

## Introduction

Thyroid surgery has been performed for thousands of years and still remains one of the more common head and neck procedures (1). With the modernization of thyroid surgery, the mortality rate has significantly dropped to less than 1% compared to 40% when in 1850, the French Medical Society banned these operations (2). The open neck approach that Kocher fathered is still used today, while newer methods to address thyroid nodules have evolved to address specific shortcomings. Such methods involve accessing the thyroid remotely with an endoscope in order to avoid a neck incision, or by minimally invasive techniques such as thermal ablation to avoid removing the thyroid gland at all. Regardless of the surgical approach, complications still exist.

In the hands of an experienced and high volume thyroid surgeon, complication rates may vary by disease pathology. Intraoperative injury to the recurrent laryngeal nerve (RLN) is one of the dreaded complications that can occur with open surgical approaches and less commonly with thermal ablation. Symptoms of unilateral vocal cord paralysis may include hoarseness, choking, dysphagia, and dysphonia with unilateral vocal cord paralysis, or symptoms of airway obstruction and stridor with bilateral vocal cord paralysis (3–6). Changes in the quality of voice or swallowing can significantly diminish a patient’s ability to work, socialize and perform many activities of daily living, thus emphasizing the importance of careful handling of the RLN (7, 8).

For open neck approaches to the thyroid, approximately 1 in 10 patients experience temporary RLN injury after surgery, with longer lasting permanent voice problems in up to 1 in 25 (9). Other contributing factors which may lead to temporary dysphonia post-surgery include laryngeal irritation, edema or injury from intubation. Injury to the external branch of the superior laryngeal nerve can also impair a patient’s quality of life by reducing the pitch and projection of the voice (10). For thermal ablation, namely radiofrequency ablation (RFA), injury to the RLN can be temporary or less frequently permanent (11–13). Voice changes are less frequently noted (0.94%, 21/2245) in benign nodules treated with RFA compared with recurrent thyroid cancer (7.95%, 14/176) (11). The overall complication rate in RFA treated primary benign nodules is 2.11% compared with 10.98% for malignant thyroid nodules (11).

In this paper, we review the surgical and nonsurgical techniques for minimizing and treating post-operative dysphonia caused by an open approach or remote access thyroidectomy as well as minimally invasive thermal ablation (namely radiofrequency ablation). In addition, we review how intraoperative electromyographic (EMG) changes correlate to post-operative voice changes, and how to treat weakness of the recurrent laryngeal nerve.

## Practice Recommendations to Minimize Dysphonia Post Thyroid Surgery

### Visualization of the Nerve

The gold standard approach for preventing inadvertent injury to the RLN during thyroid surgery has always been meticulous dissection of the nerve in order to visualize its entry into the cricothyroid joint and/or course (14). Many prospective studies have confirmed Lahey’s observation in 1938 (15) that clear visualization of the RLN during thyroid surgery significantly lowers the incidence of nerve injury compared to surgery without visualization (16–20). Yet the best method to prevent nerve injury has been greatly debated. Even with a visually intact RLN, function can been compromised (21). Generally, the rate of RLN paralysis is low in experienced hands for open neck approaches (temporary injury 2%–8%, permanent in 0.5%–3%), however the rate tends to be higher for malignant thyroid disease, enlarged goiters, Graves’ disease, re-operative cases, anatomic variability and surgeon’s inexperience (22–24).

Most often, surgeons take a lateral approach for dissecting the thyroid in order to identify the RLN. Fixed landmarks, such as the cricothyroid joint, is a helpful guide for localizing the entry point of the RLN into the larynx. In certain situations a medial approach can be advantageous in cases with a nonrecurrent RLN, a large Tubercle of Zuckerkandl, or in cases with extrathyroidal extension of cancer along the distal RLN segment (with the exception of cricothyroid junction involvement), or large goiters (25). This approach allows for early and direct access to the RLN once the isthmus is divided and Berry’s ligament is exposed (25). Care must be taken to avoid inadvertent injury when localizing the RLN with the medial approach since up to 80% of the time, the RLN is located superficial to Berry’s ligament, and below it 15% of the time (26).

### Intraoperative Nerve Monitoring

Over the past few decades, intraoperative nerve monitoring (IONM) has gained acceptance as an adjunct to nerve visualization during thyroid surgery and is used by most high volume thyroid surgeons in North America (27). The American Academy of Otolaryngology – Head and Neck Surgery guidelines have recommended its use, and it has become the standard of care at most institutions. Regardless of whether the surgeon has high-volume or low-volume experience, most would argue that IONM can aid the surgeon through difficult anatomical situations should the surgical field be impaired by bleeding, scarring or tumor infiltration that may complicate a clean dissection. However, IONM does not replace knowledge of the head and neck anatomy.

Whether IONM conveys a real advantage over no monitoring at all continues to be debated by endocrine surgeons (3, 14, 22, 23, 28–31). Barczynski et al. and others have demonstrated statistically lower rates for transient RLN paralysis with IONM compared with visualization alone (32). Whereas, Higgins et al., did not report a statistically significant difference in the overall incidence of true vocal fold palsy (3.52%) for IONM compared with (3.12%) for nerve identification alone (31). In a Cochrane analysis, the rates of permanent RLN palsy (RR 0.77, 95% CI 0.33 to 1.77; *P* = 0.54; 4 trials; 2895 nerves at risk; very low-certainty evidence) and transient RLN palsy (RR 0.62, 95% CI 0.35 to 1.08; *P* = 0.09; 4 trials; 2895 nerves at risk; very low-certainty evidence) when IONM was compared with nerve visualization alone did not conclude firm evidence showing an advantage or disadvantage for prevention of RLN injury (23). However, in Zheng’s meta-analysis of over 12 different trials which included 36,487 participants, IONM was found to decrease the risk of transient RLN palsy, but had no significant effect on the permanent rate of injury (32).

The most widely available IONM system is one where two surface electrodes embedded in the endotracheal tube are positioned so they contact the vocal folds during intubation. This allows the surgeon to intermittently monitor the RLN or the vagus nerve with a handheld probe once the nerve has been surgically isolated (3, 27). This system is useful for confirming the localization of the RLN, for identifying whether there is a loss of signal from nerve injury, and to guide decision making for whether to stage the surgery in the case of an ipsilateral loss of signal during a bilateral procedure (3, 27, 33). Intermittent IONM has its limitations, namely the need to maintain constant contact with the surface electrodes on the endotracheal tube and the vocal folds to obtain an accurate EMG recording. Should the endotracheal tube shift during surgical manipulation, a false decrease or loss in the EMG signal can result. Once the surgery is in progress, it can be disruptive for the surgeon or the anesthetist to reposition the tube, particularly during remote access surgeries such as the transoral robotic approach (34). Various newer approaches have emerged to circumvent these limitations (33, 34).

Continuous IONM has evolved to circumvent the limitations of intermittent IONM so that nerve monitoring can occur continuously and in real time. It is beneficial for the early detection and prevention of thermal or traction injuries, thus allowing the surgeon to take immediate action if a change in the EMG is noted (28, 29). Some studies have noted improved outcomes with continuous monitoring relative to intermittent monitoring, which has spurred adoption in some centers (35). In a comparative study, Schneider et al., examined a total of 6029 patients, of whom 3139 underwent continuous and 2890 intermittent IONM (35). Continuous IONM independently reduced early postoperative vocal cord palsy 1.8-fold (OR 0.56) and permanent vocal cord palsy 29.4-fold (OR 0.034) compared with intermittent IONM (35). Early postoperative vocal cord palsies were 17.9-fold less likely to become permanent with continuous IONM than intermittent IONM, demonstrating the advantage for using continuous monitoring in the right clinical scenario (35).

### Hemostasis with Energy Based Devices

With the modernization of thyroid surgery, advances have included the use of energy-based devices (EBD) as an extension from the “clamp-and-tie” technique that Theodore Kocher fathered in the 19th century. Today, hemostasis can be achieved in multiple ways: clamp-and-tie, electrocautery (monopoloar or bipolar), with hemostatic clips, and more advanced EBD that use thermal, ferromagnetic, or ultrasonic energy to ligate, seal and dissect tissue (36). Even though more advanced techniques with EBD have demonstrated reduced pain, wound drainage, decreased rates of neck hematoma and even hypocalcaemia (37–40), the results for EBD are inconsistent in terms of rates of RLN injury compared with conventional approaches (41, 42). Several studies have demonstrated that traction and thermal injury are the first and second most common causes of iatrogenic RLN injury during thyroidectomy (39, 43, 44). As one would expect, the use of EBDs can generate high temperatures that can spread to critical structures such as the RLN causing indirect thermal spread or direct thermal injury (36). Thus, various guidelines are published to ensure that surgeons maintain a safe distance between the activated EBD tip and the surrounding soft tissue (36). It is also recommended that enough time lapses for the tip or blade to cool sufficiently before using the device to dissect tissue or work close to the RLN.

Depending on whether monopolar electrocautery or bipolar cautery is used, a safe distance must be maintained in order to protect critical structures. Care should be taken when using monopolar electrocautery adjacent to the RLN given that thermal spread is diffuse. In a porcine model, Wu et al. demonstrated that an activation distance of 5 mm is required to maintain safety with a cooling time of 1 s for monopolar electrocautery set at 15 Watts (45). For bipolar cautery the recommendations differ since the current is confined to the tissue between the two arms of the tines (forcep-shaped electrode). Wu et al. reported an activation distance of 3 mm from critical structures with a 1 s cooling time set at 30 Watts (45). For advanced bipolar EBDs such as the LigaSure Small Jaw and Ligasure Exact Dissector, Dionigo et al, recommend a safe activation distance of 2 mm with a 2-second interval for cooling the instrument tip before further dissecting tissue (46). Similar distances are noted for EBDs that deliver energy in the form of ultrasonic vibrations (such as the Harmonic) or Ferromagnetic energy (47). Regardless of the EBD used, surgeons should be aware of the device specific recommended distance needed for a safe dissection. The perception of distance may be altered in remote access surgery where the surgical field is closed and visualized via endoscope compared with traditional open neck approaches but is key for minimizing thermal injury and for improving voice outcomes.

Thermal injury to the RLN is more detrimental to voice outcomes than mechanical injury cause by traction or compression. Studies have demonstrated that thermal injury can cause irreversible changes to the nerve, damaging the endoneurium with temperatures at little as 60 Celsius (48). Most EBDs reach temperatures that exceed 200 Celsius when they are activated, and more than 350 Celsius with monopolar electrocautery (36). Additional care should be taken to avoid inadvertent injury to nearby structures when operating in a wet surgical field, as high temperature steam can be generated if the EBD is activated (49). Furthermore, surgeons should be aware and cautious when using EBDs as dissectors post-activation particularly during endoscopic approaches where the field of view can be limited.

Despite meticulous dissection along tissue planes, a slow oozing type of bleeding can still occur often around neural structures that are nourished by smaller vessels. The use of clips, cautery or ties adjacent to the nerve can pose risk. For this reason, various hemostatic agents have been developed in the form of a gel or patch to help mitigate this risk of a hematoma and nerve injury with cauterization. The use of such hemostatic agents, such as topical gels and patches have been shown to be a safe practice and does not put the RLN at risk. In a meta-analysis comparing the use of a topical hemostatic patch or gel to conventional methods for hemostasis, no significant difference between groups (patch 95% CI, 0.28, 5.52; gel 95% CI, 0.20, 2.47) were found in terms of risk to RLN injury (50).

## Practice Recommendations to Minimize Dysphonia Post Thermal Ablation with RFA

The true rate of RLN injury post thermal ablation is not known since studies examining pre- and post-procedure laryngoscopy are lacking. However, the overall rate of transient or permanent voice change following RFA is 1.44% based on subjective voice assessment (11, 51).

Since RFA is done in an outpatient setting with local anesthesia, temporary voice changes can be noted either during radiofrequency ablation (RFA) or immediately after the procedure. The most important approach for mitigating this potential complication is to carefully map the location of the target nodule by ultrasound in relation to the “danger triangle”; which is localized posterior to the thyroid capsule and adjacent to the trachea where the RLN runs (52). The only study which specifically examined the effects of RFA along the posterior thyroid capsule is found in a recent prospective study. Sinclair et al. demonstrated in thirteen benign nodules that abutted the posterior thyroid capsule, that RFA could safety be performed at a power of up to 40 Watts without laryngeal adductor reflex (LAR) amplitude changes measured by continuous intraoperative neuromonitoring (CIONM) (13). No significant difference between pre- and postoperative laryngoscopy and voice assessments were noted, and after 12 month follow-up, regrowth was not noted at the posterior aspect of the nodule (13). For nodules that extend beyond the posterior thyroid capsule, or for nodules that lack a cuff of normal thyroid tissue, surgery should be considered instead of RFA due to the risk to the RLN and the lack of tissue to buffer thermal spread, especially if the nodule is malignant.

While RFA can be completed under general anesthesia with nerve monitoring, most operators feel that local sedation offers an improved safety profile. One of the benefits of an outpatient procedure is the real-time feedback that patients can give the operator in terms of pain control, change in voice or breathing. An important way to monitor for thermal damage to the RLN during ablation is to maintain communication with the patient during the procedure. Prior to the thermal ablation, perithyroidal lidocaine is injected around the anterior or superficial thyroid capsule to reduce pain. By avoiding anesthesia of the deeper tissues, if pain is experienced during the procedure, it can be an early sign of thermal propagation to the surrounding tissue and the electrode can be moved and then power can be reduced by 5 to 10 Watts before proceeding further (12, 53). If patients note changes to their voice immediately post-procedure, most often it is transient and can be treated with a short course of Prednisone during their recovery period. Furthermore, when using RFA to ablate bilateral thyroid nodules, care should be taken to monitor for voice changes to avoid bilateral RLN paresis. The operator should have a low threshold for staging bilateral procedures should dysphonia be noted during RFA.

Another useful approach for minimizing injury to the surrounding tissue (ie. RLN) prior to ablation is hydro-dissection with 5% dextrose solution along the plane between the target tumor and adjacent critical structures. This creates a thermal barrier as well as a “heat-sink effect” which allows for heat to escape during ablation (54). Dextrose solution is the fluid of choice since it is iso-molar (252 mOsmol/L) and non-ionic in composition, thus safer than normal saline which is anionic and able to conduct electricity. If thermal injury is suspected post-procedure, Lee et al. describe injecting cold 5% dextrose solution into the tracheoesophageal groove post-ablation as an effective method for cooling the tissues and for reducing heat conduction to surrounding structures (55). Compression of the neck is not recommended since it may enhance heat conduction to the surrounding tissue.

The safest technique described for performing RFA is the “moving shot technique”, which involves alternating the position of the electrode in the nodule so each theoretical sub-unit is ablated. This limits the frictional heat generated from the electrode tip by intermittent active movement and limits the time of ablation for each subunit to a few seconds compared to a fixed approach used in organs such as the liver (54). By using the trans-isthmic approach, which refers to insertion of a RF electrode through the isthmus, this allows the operator to be able to pivot from a midline to lateral direction to access different angles depending on whether the nodule is situated in the right or left thyroid gland, and enables the operator to ensure that the contents of the “danger triangle” can be protected (54). By inserting the probe in the midline, it allows the operator to effectively monitor the electrode tip using ultrasound guidance and to stabilize the needle should a patient talk or cough during the procedure.

Although multiple sized electrodes are available for targeting different organs, it is important to use thyroid-dedicated internally cooled electrodes for RFA given the superficial and intricate anatomy of the thyroid and neck. Generally, for the thyroid, RFA consists of a 7-cm internally cooled electro (shaft length) with an 18 or 19-gauge active tip that measures either 5 mm, 7 mm, 10 mm or 15 mm) with typical workhorse tips sizes of 7 mm or 10 mm depending on the patient population (54, 56). Depending on where the nodule is located, small active tips (3.8 mm) can allow for a more precise treatment limiting collateral thermal spread to nearby structures (54).

## Correlation Between Intraoperative Electrophysiological Change of RLN/EBSLN and Voice Impairment in Thyroid Surgery

If a loss of signal or a decrease in the EMG amplitude is noted intraoperatively and equipment malfunction or neuromuscular blockage has been ruled out, careful stimulation of the nerve with the probe from distal (the laryngeal nerve entry site) to proximal is recommended to identify the site of neuropraxic injury. This is typically done for open neck procedures compared with closed remote access techniques. Generally, nerve injuries identified through intraoperative neural monitoring are divided into two types: Type I (segmental) or Type II (global) injury (27). Segmental injuries can be corrected if a section of the nerve is entrapped with a clip or a suture, however for global type injuries, where all segments are nonconductive, an intralaryngeal focus is more likely.

Several papers have looked at the correlation between intraoperative EMG responses and postoperative vocal cord mobility. The International Neural Monitoring Study Group Guideline describes an impending adverse EMG even defined as an amplitude decrease of >50% of the initial baseline value, and an adverse EMG event is defined as an amplitude of <100 uV (14, 27). A true negative test is one where the EMG response is within normal limits at the end of surgery with intact vocal cord mobility postoperatively. Several studies have demonstrated that when the EMG has a robust response at the end of surgery, intact post-operative vocal fold functioning (the negative predictive value) is more than 95% (57–64). In a more recent review, Schneider et al., describe an even higher NPV for RLN palsy (97.3%–99.8%) when intermittent, and continuous (99.8%–100%) IONM is used (64). In contrast, if a loss of signal is noted on EMG at the end of surgery combined with immediate post-operative vocal cord paralysis, the outcome is a true positive test (27). However, for PPV, the rate is more variable due to transient versus permanent paresis, with a range of 37.8%–80.5% for intermittent, and 99.8%–100% for continuous IONM (63, 64). These values still translate into excellent outcomes with low rates of post-operative vocal cord palsy in experienced surgical hands, with early transient palsy 0.8%–10.5% for intermittent, and 2.6%–2.9% for continuous IONM (64). For permanent palsy, the rates are lower (0.2%–1.5% for intermittent, and 0%–1.0% for continuous IONM) (64). As a general rule, if there is an intact IONM signal at the end of the case, any post-operative voice changes are unlikely to be related to permanent RLN dysfunction.

## Voice and Laryngeal Assessment Before and After Thyroid Surgery and Thermal Ablation

It is standard of care to assess the patient’s voice prior to thyroid surgery or thermal ablation to establish baseline function that can be compared post-surgically. As part of the history, patients should be asked about subjective changes in their pitch, loudness, quality or power of their voice (65). Validated standardized methods can also be employed, such as the Voice Handicap Index. This 30-item questionnaire determines the degree of voice impairment and has been used as a tool to determine pre- and postoperative voice status after thyroid surgery (66, 67). During physical exam, all patients with subjective voice changes should receive a preoperative glottic exam to ensure normal baseline glottic functioning. Approximately 1% of patients with benign thyroid disease have vocal fold paresis or paralysis and in up to 8% of patients with malignant thyroid disease who have not undergone prior thyroid, neck or chest surgery (68). Given that a preoperatively diagnosis for vocal fold paralysis is suspicious for thyroid cancer, such as finding may alter the surgeon’s approach to surgical resection as well as how the patient is counselled preoperatively regarding expected voice outcomes.

The most commonly available method for examining the larynx is by flexible nasopharyngoscopy. More traditional methods involve the mirror exam, but have increasingly been replaced by flexible nasopharyngoscopy. Video-strobo-laryngoscopy (VSL) is another way to do a detailed functional examination of the larynx, however is less readily available except in more laryngology-based practices.

## Speech Therapy and Injection Laryngoplasty for Post-Thyroid Procedure Dysphonia (PTD)

**Figure 1:**
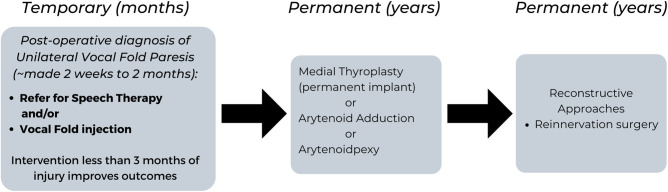
Flow Chart summarizing the Management for Temporary and Permanent Post-Operative Dysphonia due to Unilateral Vocal Paresis.

When a patient presents with complains of breathiness, dyspnea and/or mild dysphagia post-thyroid surgery, and vocal fold immobility has been confirmed on flexible nasopharyngoscopy, several options that can be offered. The management of symptomatic patients post-thyroid surgery varies from patient to patient but can include speech therapy, vocal fold injection, thyroplasty, reconstructive approaches for unilateral vocal fold paralysis and tracheotomy for bilateral vocal fold paralysis (**Figure 1**).

The clinical practice guidelines for improving voice outcomes after thyroid surgery recommend that assessment of voice is done within 2 weeks to 2 months post-thyroid surgery by the surgeon (65). If voice changes are noted, a laryngeal mirror or flexible nasopharyngoscopy is done to document whether vocal fold paresis is present. If the surgeon does not have the equipment to assess the vocal folds, a referral is made to an Otolaryngologist – Head and Neck surgeon for further management. Once a diagnosis of unilateral vocal fold paresis is made or suspected, shared decision making with the patient regarding the next steps of management is made. Of note, the earlier the intervention with speech therapy and/or vocal fold injection (less than 3 months), the better the long-term outcomes in voice and swallowing for the patient (65).

Speech therapy focuses on rehabilitation of vocal fold approximation using behavioral approaches. The goal of voice therapy is to improve glottic closure by strengthening the intrinsic muscles of the larynx, rather than developing abdominal support for breathing and supraglottic hyperfunction (65). Procedural approaches include vocal fold injection, which is a temporary method to augment the immobile vocal fold to a more midline position. This outpatient procedure can improve laryngeal function by improving glottic closure during the recovery period, while also reducing the likelihood for permanent medialization laryngoplasty (69). In a retrospective review, Yung et al. demonstrated the benefit of temporary injection medialization in 19 patients with unilateral fold paresis compared with 35 patients who were managed conservatively (69). Those patients injected were significantly less likely to require permanent medialization laryngoplasty (*p* = 0.0131). In terms of the timing for injection medialization, Friedman et al., demonstrated the earlier the intervention, the better (70). In patients that had early injection medialization (less than 6 months from the time of injury to medialization), 62% (20/32) maintained an adequate voice obviating the need for open neck surgical reconstruction, whereas 100% (3/3) that underwent late injection (more than 6 months post paralysis) required surgical reconstruction (*P* = 0.03) (70).

For permanent unilateral vocal fold paralysis, medialization thyroplasty is an option to mobilize the vocal fold to a midline position through a small transcervical incision (71). Other permanent methods involve manipulating the laryngeal cartilage to perform an arytenoid adduction or arytenopexy (72, 73). These more advanced procedures involve an operating room setting and are generally done by surgeons with a laryngology based practice. Laryngeal reinnervation is another advanced procedure which is less commonly done, and typically performed by connecting the ansa cervicalis and the recipient RLN (74). Although there is a delay in voice improvement with this technique, it can partially improve the position of the vocal fold and provide bulk so long-term denervation atrophy of the laryngeal muscles is avoided (74). Often, the delay in voice improvement can be bridged with injection laryngoplasty. Depending on what was noted intraoperatively in terms of tumor involvement around the RLN, or transection of the nerve, decisions can be made to go directly to permanent solutions (such as medial thyroplasty, arytenoid adduction, arytenopexy or reinnervation surgery) if the nerve is deemed unlikely to recover.

## Conclusion

Post-operative dysphonia has serious implications and consequences to patients, and thus, every effort should be taken to mitigate this complication. Various surgical and nonsurgical approaches can be taken reduce vocal fold injury during thyroid surgery as well as during thermal ablation. In symptomatic patients with RLN injury, various treatment options can be offered which range in behaviour therapy to more surgical type approaches to improve their quality of life.
